# BMDExpress: a software tool for the benchmark dose analyses of genomic data

**DOI:** 10.1186/1471-2164-8-387

**Published:** 2007-10-25

**Authors:** Longlong Yang, Bruce C Allen, Russell S Thomas

**Affiliations:** 1 The Hamner Institutes for Health Sciences, 6 Davis Drive, Research Triangle Park, NC 27709-2137, USA; 2 Bruce Allen Consulting, 101 Corbin Hill Circle, Chapel Hill, NC 27514, USA

## Abstract

**Background:**

Dose-dependent processes are common within biological systems and include phenotypic changes following exposures to both endogenous and xenobiotic molecules. The use of microarray technology to explore the molecular signals that underlie these dose-dependent processes has become increasingly common; however, the number of software tools for quantitatively analyzing and interpreting dose-response microarray data has been limited.

**Results:**

We have developed BMDExpress, a Java application that combines traditional benchmark dose methods with gene ontology classification in the analysis of dose-response data from microarray experiments. The software application is designed to perform a stepwise analysis beginning with a one-way analysis of variance to identify the subset of genes that demonstrate significant dose-response behavior. The second step of the analysis involves fitting the gene expression data to a selection of standard statistical models (linear, 2° polynomial, 3° polynomial, and power models) and selecting the model that best describes the data with the least amount of complexity. The model is then used to estimate the benchmark dose at which the expression of the gene significantly deviates from that observed in control animals. Finally, the software application summarizes the statistical modeling results by matching each gene to its corresponding gene ontology categories and calculating summary values that characterize the dose-dependent behavior for each biological process and molecular function. As a result, the summary values represent the dose levels at which genes in the corresponding cellular process show transcriptional changes.

**Conclusion:**

The application of microarray technology together with the BMDExpress software tool represents a useful combination in characterizing dose-dependent transcriptional changes in biological systems. The software allows users to efficiently analyze large dose-response microarray studies and identify reference doses at which particular cellular processes are altered. The software is freely available at  and is distributed under the MIT Public License.

## Background

The endogenous control and external perturbation of biological processes are inherently dose-dependent. Examples include developmental events that require gradients of growth factor concentrations [1], zonation in the liver due to differences in oxygen and nutrient concentration [2], the pharmacological inhibition of key proteins in disease [3], and the toxic effects of environmental chemicals [4]. Without a proper understanding of the dose-response characteristics, the molecular mechanisms underlying the regulation or perturbation of these biological processes would remain unknown.

Microarray technology has been broadly accepted as an efficient and reproducible way to explore the gene expression changes involved in the regulation of biological processes. The ability to survey thousands of genes allows a comprehensive assessment of the transcriptional changes involved in specific cellular events. Bioinformatic methods have been developed to interpret these changes by applying standardized functional annotations to each gene and identifying whether certain biological processes or molecular functions are over- or under-represented [5-10]. This approach has been referred to as a gene ontology (GO) enrichment analysis and allows large lists of transcriptional alterations to be distilled down into changes in cellular processes such as the immune response, DNA repair, apoptosis, etc.

To quantitatively assess the dose-response behavior of endogenous molecules and environmental chemicals, benchmark dose (BMD) methods have been employed to estimate reference doses [11-13]. In the BMD method, dose-response data for the biological effect is fit with a statistical model and a BMD is identified that results in a defined level of response over that observed in control populations. The BMD method has been used extensively by regulatory agencies to set standards for human health effects [14,15].

A method for integration of BMD calculations with GO classification analysis in the examination of microarray dose-response data has recently been developed [16]. The combination of microarray technology with these analysis methods results in a unique bioinformatic tool that provides both a comprehensive survey of transcriptional changes together with dose estimates at which different cellular processes are altered based on a defined increase in response. In this application note, we describe the development and availability of a user-friendly software tool that integrates these standard methods in the analysis of microarray dose-response data.

## Implementation

BMDExpress was written in the Java programming language with a Swing graphical user interface. The application requires a Java Runtime Environment of 1.6.0 or newer. Model fitting to the dose-response data is performed using a dynamic link library (DLL) written in C and FORTRAN that are called using a Java Native Interface. The DLL was written using source code modified from the BMDS software application developed by the U.S. Environmental Protection Agency [17]. In mapping the Affymetrix probe identifiers to corresponding GO categories, the software application queries a client-accessible MySQL database that resides at The Hamner Institutes. The database is constructed using annotations provided by NetAffx [18] and the Gene Ontology Consortium [19]. The database is updated weekly to ensure the annotations are current. At the present time, only Affymetrix microarrays are supported by BMDExpress and include the following: Human (HG_Focus, HG_U133A, HG-U133A_2, and HG-U133_Plus_2); Mouse (MG_U74A, MG_U74Av2, MOE430A, MOE430B, Mouse430A_2, and Mouse430_2); Rat (RAE230A, RAE230B, Rat230_2, and RG_U34A); Drosophila (DrosGenome1 and Drosophila_2); and Zebrafish (Zebrafish Genome).

## Results and Discussion

The BMDExpress software is designed to perform a stepwise analysis on dose-response microarray data that combines standard BMD methods with GO classification analysis. The program has a series of interfaces that guide the user through the analysis process.

### Data Import

**Figure 1:**
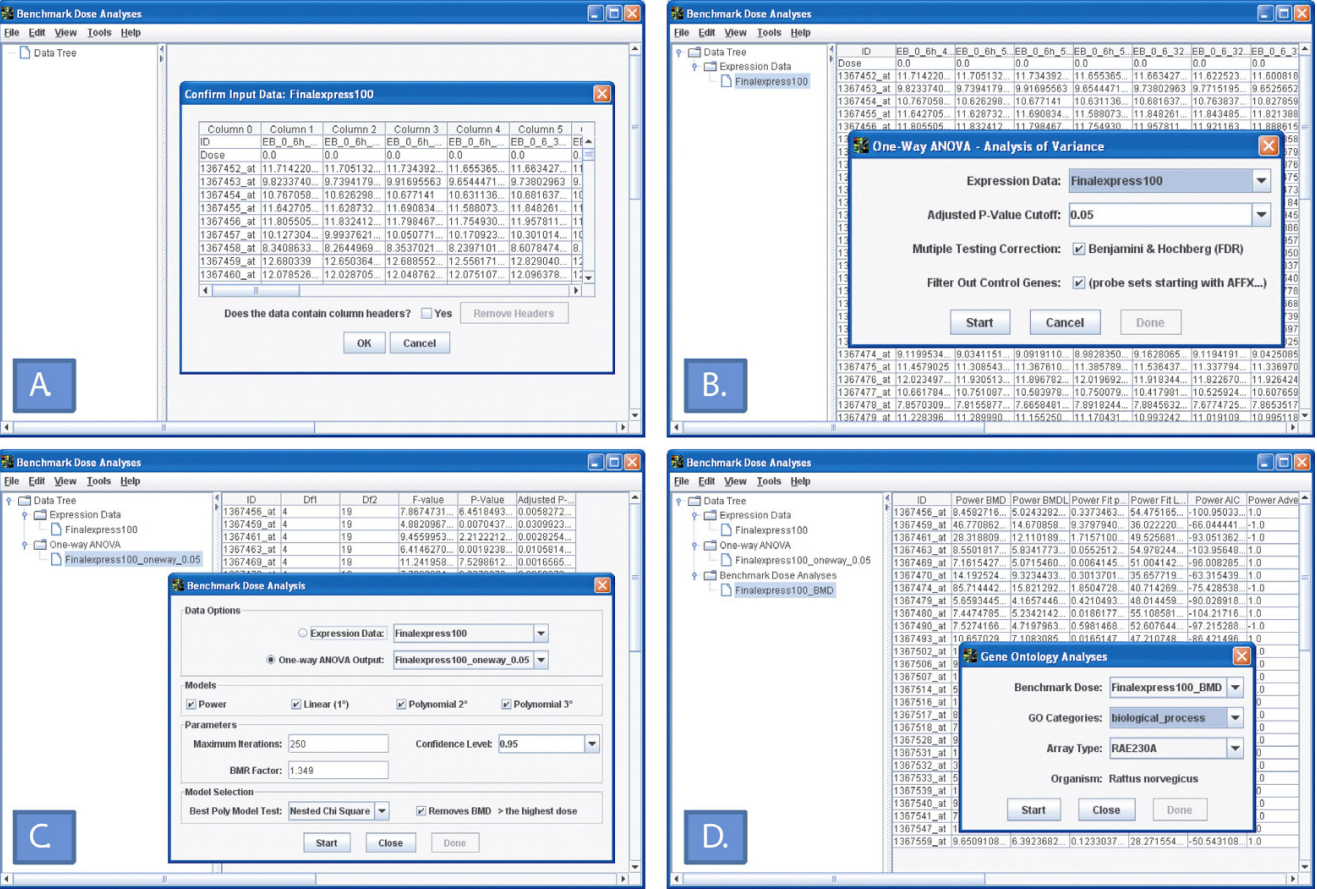
Screen shots of BMDExpress software application. The software uses standard drop-down windows for user interaction and the left-hand side of the main window contains a tree structure that outlines progress through the analysis process. (A) Interface for data import. The data is previewed in table format and if the input data has column headers, the user is provided the option to remove headers within the software interface. (B) Interface for pre-filtering data using a one-way ANOVA. The user is allowed to input an adjusted *p*-cutoff and select whether to filter out the various control genes (e.g., BioB, BioC, etc.) present on an Affymetrix array. (C) Interface for benchmark dose analysis. The user is allowed to select the type of statistical models and parameters used in the analysis and provided options on the process of selecting the best model. (D) Interface for gene ontology analysis. The BMD values from the previous step in the analysis process are used as input for the gene ontology analysis. The organism is automatically detected by the software based on the probe set identifiers. The user is allowed to select the class of gene ontology categories for the analysis with either biological process, molecular function, cell component, or universal (all three) as options.

The first step in using BMDExpress is to load the gene expression data from a data file (Fig. 1A). The data file must be in tab-delimited format with plain text. Each column in the data matrix should be the gene expression values for an individual sample and the first row should include the dose at which that sample was treated. If the data has an extra row of column headers, the program provides an option for removing them. An example data file is included in the software installation (Finalexpress100.txt).

### One-Way ANOVA

The number of probe sets on a standard Affymetrix microarray is relatively large and in the typical experiment, most probe sets are not significantly altered by the experimental treatment. As a result, an initial probe set selection process is recommended to reduce both the computational requirements and the variability in the final analysis by selecting probe sets that show significant dose-response behavior. The probe set selection process consists of a one-way analysis of variance (ANOVA) together with a false discovery rate correction for multiple comparisons [20] (Fig. 1B). The user is allowed to input a cutoff based on the adjusted *p*-value and may choose whether to filter out the various control genes (e.g., BioB, BioC, etc.) present on an Affymetrix array. The output of the one-way ANOVA lists the probe set identifier, the associated degrees of freedom, the F-value, the *p*-value, and the adjusted *p*-value. The user can export the list to a tab-delimited file by right-clicking on the corresponding node in the data tree within the left-hand window.

### Benchmark Dose Analyses

The generic definition of a BMD is the dose or concentration of a substance that corresponds to a specified level of response above or below that observed in a control or background population. The specified level of response within this definition is referred to as the benchmark response (BMR) and a statistical lower confidence bound on the BMD (BMDL) has been typically used by regulatory agencies to set safe levels of exposure. In BMDExpress, the identification of the BMD involves fitting the gene expression data to a selection of statistical models (linear, 2° polynomial, 3° polynomial, and power models) and selecting the model that best describes the data with the least amount of complexity (Fig. 1C). The user is allowed to select which statistical models they would like to fit to the data. By default, the power model and the three polynomial models are selected. However, the user can select a single model or any combination of two or more models. The user should note that depending on the number of doses in the study, the 3° polynomial may not be appropriate. In addition to model selection, the user is allowed to modify several critical parameters associated with the BMD analyses including the maximum number of iterations, the BMR, and the confidence level for calculation of the BMDL. The maximum number of iterations is a convergence criteria for the model fitting. The BMR is the number of standard deviations at which the BMD is defined. As a default, a BMR of 1.349 is provided. To derive this value, a normal distribution was assumed for control animals and it was assumed, *a priori*, that the changes in expression could occur in either tail, with a 1% chance of that occurring in the absence of exposure (0.5% in each tail). A BMR of 1.349 is the amount required to shift the mean response of the control distribution such that the treated distribution contains 11% in a single tail, i.e., a 10% increase over the assumed background rate of response. The 10% value for the shift in the tail area of the distribution is standard for BMD analysis [16,21]. The confidence level is the statistical lower confidence limit applied to BMD estimated by the model. The resultant lower bound on the BMD is the BMDL and is a conservative estimate of the dose at which the particular gene is altered.

In processing the model output, the user is allowed to choose the method for selecting which model best describes the data with the least amount of complexity. In the first method, a nested likelihood ratio test can be used to select among the linear, 2° polynomial, and 3° polynomial models followed by an Akaike information criterion (AIC) comparison [22] between the best nested model and the power model (i.e., the model with the lowest AIC is selected). In the second method, a completely AIC-based selection process is performed. Finally, the user is allowed to remove probe sets where the BMD is greater than the highest dose. This option was provided to avoid model extrapolation.

In the output from the BMD analyses, the probe set identifier is provided with the best overall model together with selected values for each of the statistical models evaluated. These include the BMD, the BMDL, the fit *p*-value from the likelihood ratio test, the log-likelihood value for the fit of the model, the AIC, and the direction of the response (i.e., increased expression or decreased expression). On an average computer, 10 probe sets can be processed per minute.

### Gene Ontology Analyses

The BMD values from the previous step in the analysis process are used as input for the GO analyses (Fig. 1D). In the GO analyses, the probe set identifiers are combined into unique genes based on their NCBI Entrez Gene identifiers. When two or more probe sets are associated with a single gene, the BMDs for the individual probe sets are averaged to obtain a single value. The Entrez Gene identifiers are then matched to their corresponding biological process, molecular function, and cellular component GO categories. The program returns a wide range of summary values representing the central tendencies and associated variability of the BMD and BMDL values for the genes within each category (Table 1).

**Table 1:** Output from BMDExpress Characterizing the Dose-dependent Behavior of Each GO Category

Output from GO Analysis	Description of Output
GO Level	Level in the hierarchy of the GO category
GO Term Name	Official name of the GO category
All Genes	Total number of genes on the array assigned to the GO category
Genes from BMD Analysis	Number of genes from BMD analyses in the GO category
Percentage	Percentage of the total number of genes in GO category used in BMD analysis
Gene IDs	Entrez Gene identifiers in GO category based on Affymetrix probe set identifiers from BMD analysis
Probe IDs	Affymetrix probe set identifiers from BMD analysis
BMD Mean	Mean BMD for the genes in GO category
BMD Median	Median BMD for the genes in GO category
BMD Minimum	Minimum BMD for the genes in GO category
BMD SD	Standard deviation of BMD for the genes in GO category
BMD wMean	Weighted mean BMD for the genes in GO category (weighted by fit *p*-value)
BMD wSD	Standard deviation of the weighted mean BMD for the genes in GO category (weighted by fit *p*-value)
BMDL Mean	Mean BMDL for the genes in GO category
BMDL Median	Median BMDL for the genes in GO category
BMDL Minimum	Minimum BMDL for the genes in GO category
BMDL SD	Standard deviation of the BMDL for the genes in GO category
BMDL wMean	Weighted mean BMDL for the genes in GO category (weighted by fit *p*-value)
BMDL wSD	Standard deviation of the weighted mean BMDL for the genes in GO category (weighted by fit *p*-value)
5th Percentile Index	N^th ^gene number representing the 5th percentile for all the genes in the category. The value is zero-based and a 0.5 value means that it falls between two values
BMD at 5th Percentile	BMD at the 5th percentile for all genes in the GO category (including genes with no significant dose response)
10th Percentile Index	N^th ^gene number representing the 10th percentile for all the genes in the category. The value is zero-based and a 0.5 value means that it falls between two values
BMD at 10th Percentile	BMD at the 10th percentile for all genes in the GO category (including genes with no significant dose response)
Probes with Adverse Direction Up	Number of probe sets in the GO category for which the final change in expression was in the up (i.e., increased) direction
Probes with Adverse Direction Down	Number of probe sets in the GO category for which the final change in expression was in the down (i.e., decreased) direction

### Example Analysis On Estrogenic Dose-Response in Zebrafish (Danio rerio)

To illustrate the features and functionality of BMDExpress, microarray data were taken from a study of hepatic gene expression changes in zebrafish following exposure to 17 α-ethynylestradiol (EE2) (ArrayExpress Accession No. E-TABM-105) [23]. In this study, female zebrafish were exposed to EE2 in the water at nominal concentrations of 0, 15, 40, and 100 ng/L for 24 and 168 h. Gene expression changes in the liver were then evaluated using the Affymetrix Zebrafish array to identify potential genomic biomarkers for endocrine disrupting compounds (natural and synthetic) that are released into the environment and obtain a better understanding of the biological effects of these compounds in fish. The data from the study was downloaded and normalized using robust multi-array averaging (RMA) with a log_2 _transformation [24]. The log_2 _transformed data for each time point were imported and analyzed separately using BMDExpress. The data were pre-filtered using a one-way ANOVA with a false discovery rate of 5%. A total of 1061 and 864 probe sets showed significant dose-response behavior at the 24 h and 168 h time-points, respectively. The data were then fit to linear, 2° polynomial, and power models and the best model was selected using a likelihood ratio test for the linear and 2° polynomial followed by an AIC comparison with the power model. The BMD values from the best model were then used as input for the GO analysis.

Results from the analysis at the 24 h time point showed that the most sensitive single gene was tryptophanyl-tRNA synthetase (*Wars*) (BMD = 5.74 ng/L) and the most sensitive biological process to EE2 exposure was amino acid glycosylation (BMD = 10.88 ± 1.80 ng/L) (Table 2). The four responsive genes in this category all showed similar dose-response behavior. Based on the BMD values, the genes relating to amino acid glycosylation and tryptophanyl-tRNA synthetase may be a more sensitive set of biomarkers than the current single standard biomarker of vitellogenin [25]. Changes in serum vitellogenin concentrations were only significantly altered at the 40 ng/L concentration following the 24 h exposure [23]. The regulation of protein glycosylation by sex steroids has been well established [26]. For example, the glycosylation of follicle-stimulating hormone is regulated by estrogens and androgens and affects its biological activity [27]. Another example is the glycosylation of vitellogenin itself. Vitellogenin has been shown to be a highly modified protein [28] and the post-translational changes include glycosylation by hepatocytes [29]. The glycosylation of vitellogenin provides a source of carbohydrate for the developing embryo [30] and may play a role in transport, folding, and uptake of vitellogenin [31]. Other GO categories that showed low BMD values were cell migration involved in gastrulation, monocarboxylic acid metabolism, tRNA aminoacylation, nitrogen compound metabolism, and regulation of apoptosis. At the 168 h time point, the most sensitive single gene was dihydrolipoamide dehydrogenase (*Dldh*) (BMD = 5.96 ng/L) and lipid transport was the most sensitive biological process (11.57 ± 2.54 ng/L) (Table 2). In the lipid transport category, one of the genes was vitellogenin and another was apolipoprotein A-I. Both genes have been previously shown to be transcriptionally-regulated by estrogen [25,32]. Other GO categories that showed relatively low BMD values were glycolysis, protein-DNA complex formation, and protein import.

**Table 2:** Biological process GO categories with the lowest mean BMD in zebrafish exposed to EE2

Biological Process GO Category^a^	Total Genes in Category	Genes with BMD	Mean BMD (ng/L)	Std Dev BMD	Mean BMDL (ng/L)	Minimum BMD (ng/L)	Upregulated/Downregulated Probes
24 h							
Protein amino acid glycosylation	24	4	10.88	1.80	7.56	9.47	4/0
Cell migration involved in gastrulation	28	3	12.07	0.82	8.24	11.46	0/3
Monocarboxylic acid metabolic process	28	3	12.30	5.79	8.15	7.90	5/0
tRNA aminoacylation	25	13	12.56	6.62	8.78	5.74	18/0
Nitrogen compound catabolic process	11	3	12.57	1.11	8.53	11.75	0/3
Regulation of apoptosis	45	3	12.76	2.59	8.59	11.24	2/1
Amino acid and derivative metabolic process	83	27	12.94	4.82	8.82	5.74	22/12
Amino acid metabolic process	72	26	12.99	4.91	8.85	5.74	22/11
Amino acid biosynthetic process	18	5	13.05	3.54	8.70	7.90	4/2
Amine metabolic process	99	30	13.07	4.63	8.88	5.74	24/14
168 h							
Lipid transport	10	3	11.57	2.54	8.60	8.64	1/3
Response to external stimulus	41	3	15.99	7.96	8.12	10.75	0/3
Glycolysis	33	6	19.75	10.52	13.14	9.27	2/5
Carbohydrate catabolic process	44	7	20.71	9.93	17.79	9.27	3/5
Alcohol metabolic process	61	10	21.39	12.25	13.14	9.27	3/8
Cellular macromolecule catabolic process	79	8	23.02	11.28	18.80	9.27	4/5
Protein-DNA complex assembly	30	3	23.15	16.13	9.61	12.85	1/2
Monosaccharide metabolic process	48	7	23.22	13.28	17.79	9.27	3/5
Cellular protein complex assembly	9	3	23.87	15.67	11.39	10.66	1/2
Protein import	17	3	23.87	15.67	11.39	10.66	1/2

^a^Redundant child GO categories containing the same genes and mean BMD were removed from the list.

In the original analysis of the data, the investigators used a linear model to identify genes that were significantly affected by dose together with a nonparametric stratified test for trend [23]. Pairwise comparisons between dose-levels and the control group were also performed. The investigators summarized these results using Venn diagrams showing overlap of genes between doses and used a GO enrichment analysis at each individual dose showing which processes were affected. The reanalysis of this dataset using BMDExpress provides several improvements over the standard analysis performed by the investigators in the original article. First, the statistical modeling capabilities of BMDExpress utilized the dose-response information inherent in the data and identified concentrations at which the response to the treatment was significantly changed based on the variability observed in the control animals. These reference concentrations were not limited to the specific doses used in the study unlike the analysis using the linear model and pair-wise comparisons. Second, the ability to model the dose-response curves and calculate associated confidence limits identified a set of potential biomarkers that were sensitive and robust. Third, instead of performing a GO enrichment analysis at each dose level, the analysis by BMDExpress summarized the data by showing which biological processes were the most sensitive to environmental estrogens and provided reference concentrations at which they were affected. In this manner, the analysis was able to highlight the changes in amino acid glycosylation that occur at lower concentrations than the effects on tRNA aminoacylation and apoptosis.

## Conclusion

Despite the numerous software programs for microarray data analysis, the majority do not provide any statistical modeling tools for analyzing dose-response studies. In the past, microarray dose-response studies have been typically analyzed using ANOVA with pair-wise comparisons between dose groups and the associated control. This type of analysis only identifies which genes are significantly altered at the specific doses used in the study. In BMDExpress, a statistical model is fit to the data and a dose-level is identified at which the response to the treatment is significantly different than that observed in the control animals. Using this method, the analysis is not constrained to the experimental doses and provides better use of the dose-response information [21]. The software tool then allows users to summarize the statistical modeling of the individual genes based on GO categories and characterize the dose-dependent behavior of specific cellular processes. These integrated capabilities make BMDExpress a useful tool for the dose-response analysis of microarray data.

## Availability and Requirements

Project Name: BMDExpress

Project Home Page: 

Operating Systems: Windows 2000 and Xp

Programming Languages: Java, C, FORTRAN

Other Requirements: Java 1.6.0 or higher

License: MIT Public License

Any Restrictions to Use By Non-Academics: None

## List of Abbreviations

GO, gene ontology; BMD, benchmark dose; DLL, dynamic link library; ANOVA, one-way analysis of variance; BMR, benchmark response; BMDL, BMD lower confidence limit; AIC, Akaike information criterion; EE2, 17 α-ethynylestradiol; RMA, robust multi-array averaging.

## Authors' contributions

LY created the software and drafted the manuscript. BCA consulted on the statistical modeling and analysis workflow. RST conceived and supervised the project and helped to draft the manuscript. All authors read and approved the final manuscript.
